# CD25^+^CD127^+^Foxp3^-^ Cells Represent a Major Subpopulation of CD8^+^ T Cells in the Eye Chambers of Normal Mice

**DOI:** 10.1371/journal.pone.0170021

**Published:** 2017-01-12

**Authors:** Tomasz Maślanka, Natalia Ziółkowska, Hubert Ziółkowski, Joanna Małaczewska

**Affiliations:** 1 Department of Pharmacology and Toxicology, Faculty of Veterinary Medicine, University of Warmia and Mazury, Olsztyn, Poland; 2 Department of Histology and Embryology, Faculty of Veterinary Medicine, University of Warmia and Mazury, Olsztyn, Poland; 3 Department of Microbiology and Clinical Immunology, Faculty of Veterinary Medicine, University of Warmia and Mazury, Olsztyn, Poland; Wayne State University School of Medicine, UNITED STATES

## Abstract

The aim of this study has been to determine whether eye chambers constitute part of the normal migratory pathway of naive CD4^+^ and CD8^+^ T cells in mouse and if natural CD4^+^CD25^+^Foxp3^+^ and CD8^+^CD25^+^Foxp3^+^ regulatory T cells are present within these eye compartments. To this aim, the cells obtained from aqueous humor (AH) of normal mice were phenotyped in terms of the expression CD4, CD8, CD25, CD127 and transcription factor Foxp3. The mean percentage of CD8^+^ T cells in the total AH lymphocyte population was as high as 28.69%; the mean percentage of CD8^high^ and CD8^low^ cells in this population was 34.09% and 65.91%, respectively. The presence of cells with the regulatory phenotype, i.e. CD25^+^Foxp3^+^ cells, constituted only 0.32% of CD8^+^ T cell subset. Regarding the expression of CD25, AH CD8^+^ T cells were an exceptional population in that nearly 85% of these cells expressed this molecule without concomitant Foxp3 expression. Despite having this phenotype, they should not be viewed as activated cells because most of them co-expressed CD127, which indicates that they are naive lymphocytes. With regard to the markers applied in the present research, CD8^+^CD25^+^CD127^+^Foxp3^-^ T cells represent the most numerous subset of AH CD8^+^ cells. The results suggest that eye chambers in mice are an element in the normal migratory pathway of naive CD8^+^ T cells. The study presented herein demonstrated only trace presence of CD4^+^ cells in the eye chambers, as the mean percentage of these cells was just 0.56. Such selective and specific homing of CD8^+^ and CD4^+^ cells to the eye chambers is most clearly engaged in the induction and maintenance of ocular immune privilege.

## Introduction

The eye is an immune privileged site where some immune responses are down-regulated or completely abolished to protect the delicate internal structures of the eye from the damage and permanent injury that could be consequences of strong inflammatory responses. Hori [1] distinguished three major categories of mechanisms involved in the induction and maintenance of immune privilege in the eye: these are (1) anatomical, cellular, and molecular barriers in the eye; (2) eye-derived immunological tolerance, the so-called anterior chamber-associated immune deviation (ACAID); and (3) immune suppressive intraocular microenvironment. Considering the profound role of naturally occurring CD4^+^CD25^+^Foxp3^+^ regulatory T cells (nTregs) in the maintenance of immunologic homeostasis and tolerance, a question arises whether these cells contribute to the immune privileged status of the eye. CD4^+^CD25^+^Foxp3^+^ Tregs can be divided into two principal subsets: nTregs and induced (also called inducible or adaptive) Tregs (iTregs). CD4^+^CD25^+^Foxp3^+^ nTregs develop in the thymus [2], while iTregs develop in the periphery from conventional CD4^+^ T cells [3]. It is well established that nTregs play a pivotal role in the maintenance of the balance between the tissue-damaging and protective effects of the immune response [4]. Similarly to CD4^+^ T cells, natural and induced CD8^+^CD25^+^Foxp3^+^ Tregs are also present within the pool of CD8^+^ T cells. There are reports indicating that inducible CD8^+^CD25^+^Foxp3^+^ Tregs may play an important role in immune regulation of the anterior segment of an eye [5] and in the development of ACAID [6–7]. However, the available literature lacks studies and data resolving the question whether there are naturally occurring CD8^+^CD25^+^Foxp3^+^ Tregs present within the anterior segment of an eye. Hence, one of the objectives of the current study has been to verify the hypothesis presuming that the presence of CD4^+^CD25^+^Foxp3^+^ and CD8^+^CD25^+^Foxp3^+^ nTregs within eye chambers is involved in the induction and maintenance of the immune privilege of the eye.

Only activated and effector memory T cells have been thought to access non-lymphoid tissues. In contrast, naive T cells have been assumed to recirculate exclusively between secondary lymphoid tissue via the blood and lymphatic systems. Evidence is now emerging that this view may be too simplistic and that naive T cells routinely traffic through non-lymphoid organs in a manner similar to that of memory T cells [8]. Cose et al. [9] examined the phenotype of CD4^+^ and CD8^+^ T cells in various non-lymphoid organs (i.e. skin, liver, gut, pancreas, kidney, testes and brain) and they showed that a significant proportion of T cells in these organs was phenotypically naive. These and other results [10–12] indicate that naive T cells may circulate through non-lymphoid tissues as part of their normal migratory pathway. Naive T cells can be activated and/or tolerized outside lymphoid organs, hence they may be as functionally important outside the secondary lymphoid tissue as within it [13]. Taking into consideration this fundamental shift in the current paradigm of T cells migration through different types of tissue, the second purpose of the present study has been to investigate whether the eye chambers constitute part of the normal migratory pathway of naive CD4^+^ and CD8^+^ T cells in the mouse. It should be underlined that the perusal of available literature has shown an almost complete absence of data on the occurrence of T cells in the eye chambers of healthy humans and animals. In one study no lymphocytes were detected in the aqueous humor (AH) of 3 healthy human subjects [14], while in another one the surface phenotype of lymphocytes from aqueous humor of healthy controls could not be identified because of the low cell count [15]. In turn, Avundruk et al. [16–17] determined the CD4/CD8 ratio in AH samples which were taken from patients who were operated because of senile cataract. However, this study did not reveal what percentage within the lymphocyte population was composed of CD4^+^ and CD8^+^ T cells, nor did it determine more precisely the phenotypes of these cells.

## Materials and Methods

### Animals & ethics statement

AH samples were derived from 8-week-old Balb/c mice. Mice were bred and maintained under standard lab conditions [12/12 h light/dark cycle, controlled temperature (21 +/- 2°C) and humidity (55+/- 5%), and with *ad libitum* access to food and water] in the Animal Laboratory of the Faculty of Veterinary Medicine, University of Warmia and Mazury in Olsztyn. The animals were housed and treated in accordance with the rules of the Local Ethics Commission for Animal Experiments in Olsztyn (affiliated to the National Ethics Commission for Animal Experimentation, Polish Ministry of Science and Higher Education). Mice were sacrificed specifically for the purpose of this study. Law in Poland (Act of 15 January 2015 on the Protection of Animals Used for Scientific or Educational Purposes) does not require a permit from an ethics commission to conduct experiments in which samples for research are obtained *post mortem* from animals not submitted to any procedure while alive. Mice were euthanized by asphyxiation with CO_2_.

### Flow cytometry

Preliminary studies showed that it was impossible to achieve a representative/reliable number of lymphocytes in a sample of AH obtained from one or two mice. Reliable results were attainable when AH taken from several mice was pooled into one sample. Therefore, in our study as well, a single sample consisted of AH derived from both eyes of 5 (or sometimes more) mice. After euthanasia, mice were fixed onto a styrofoam board with needles and the surface of an eye ball was rinsed with saline, after which the cornea was incised with a 2.75-mm corneal knife (Kai Medical, Gifu, Japan), AH was aspirated into a pipette (Eppendorf Research Plus 0.5–10 μl, Eppendorf, Hamburg, Germany) and transferred into FACS tubes containing fluorescence-activated cell-sorting buffer [Dulbecco's PBS devoid of Ca^2+^ and Mg^2+^ with 2% (v/v) heat-inactivated FBS (both from Sigma-Aldrich)]. The average volume of aqueous humor collected from one eye was about 4–5 μl. Peripheral blood (PB) was drawn from the inferior vena cava into heparinized tubes. Erythrocytes were removed as it was previously described [18]. Crystallizable fragment (Fc) receptors were blocked using anti-FcγR monoclonal antibody (mAb) (clone: 2.4G2) for 15 min on ice. Cells were then stained for 30 min on ice for surface antigens with fluorochrome conjugated mAbs: FITC rat anti-mouse CD4 (clone H129.19, IgG2a, κ), APC-Cy7 rat anti-mouse CD8a (clone 53–6.7, IgG2a, κ), PE-Cy7 rat anti-mouse CD25 (clone PC61, IgG1, λ). In some experiment, cells were additionally stained with PE-CF594 conjugated rat anti-mouse CD127 (clone SB/199, IgG2b, κ) and PE conjugated rat anti-mouse CD3 (clone 145-2C11, IgG1, κ; all mAbs from BD Biosciences, San Jose, USA). For intracellular Foxp3 staining, cells stained for CD4, CD8 and CD25 were washed, fixed, and permeabilized using mouse Foxp3 buffer set (BD Biosciences). Subsequently, cells were labeled for 30 min at room temperature with PE-conjugated rat anti-mouse Foxp3 mAb (clone: MF23, IgG2b, BD Biosciences). Flow cytometry analysis was performed using a FACSCanto II cytometer (BD Biosciences). The data were acquired by FACSDiva version 6.1.3 software (BD Biosciences) and analyzed by FlowJo software (Tree Star Inc., Stanford, USA). The cytometry setup and tracking beads (BD Biosciences) were used to initialize PMT settings. Unstained and single fluorochrome-stained samples were used to set fluorochrome compensation levels. The entire volume of each sample was always acquired.

### Statistical analysis

Student's unpaired t-test was used to analyze the results obtained. Data were considered statistically significant if p-value was < 0.05.

## Results and Discussion

**Fig 1:**
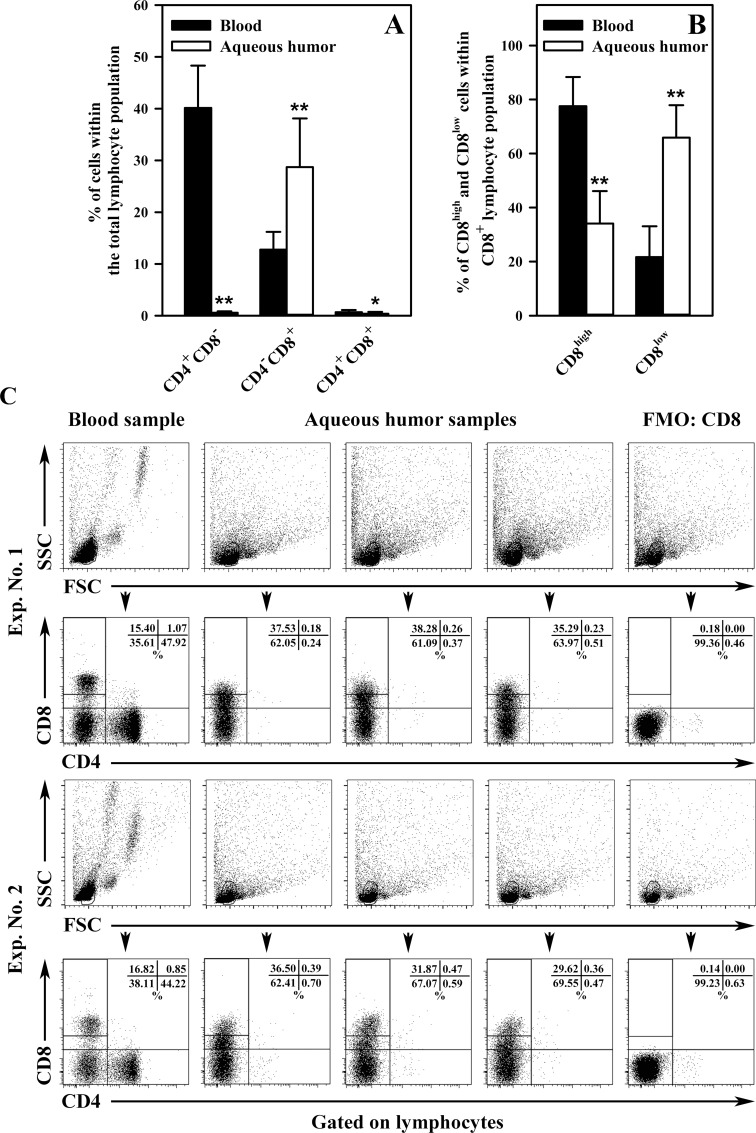
The distribution of single- and double-positive CD4^+^ and CD8^+^ T cells in mouse peripheral blood and aqueous humor. The results are expressed as percentages of CD4^+^CD8^-^, CD4^-^CD8^+^ and CD4^+^CD8^+^ T cells within the total lymphocyte population (A) and as percentages of CD8^high^ and CD8^low^ cells among the CD8^+^ T cell subset (B). Results are the mean (± SD) of 6 independent experiments. Peripheral blood samples were collected from individual mice (n = 30), whereas each aqueous humor sample consisted of cells pooled from the eyeballs of 5 (or sometimes more) mice (n = 30 samples). Examples of cytograms (dot plots) from different samples for different experiments (C). As the first step, the lymphocyte population in peripheral blood was gated on the basis of forward and side scatter (FSC and SSC, respectively; panel 1 and 3). The location of this gate served as a point of reference to set the lymphocyte gate for aqueous humor samples. CD4^+^CD8^-^, CD4^-^CD8^+^ and CD4^+^CD8^+^ T cell subsets were defined according to the expression of CD4 and CD8 within the gated lymphocyte subpopulation (panels 2 and 4). Relative to the intensity of CD8 expression, the CD8^+^ T cell population was subdivided into CD8^high^ and CD8^low^ cell subsets (panels 2 and 4). Fluorescence minus one (FMO) staining was used to confirm the gating strategy used to identify CD8-expressing cells (panels 2 and 4). *P < 0.01, **P < 0.001.

The experiments revealed only trace presence of CD4^+^CD8^-^ T cells (further referred to as CD4^+^ T cells) within the eye chambers, as the mean percentage of these cells in the total lymphocyte population was just 0.56% (± 0.28), while the value of this parameter for PB CD4^+^ T cells was 40.12 (± 8.19) (Fig 1A and 1C). The mean percentage of CD4^+^CD8^+^ double positive T cells (further referred to as DP cells) in AH and PB samples was 0.38% (± 0.36) and 0.69 (± 0.42), respectively (Fig 1A and 1C). The number of events within the CD4^+^ and DP T cell gates was too low to perform an analysis of the expression of CD25, Foxp3 and CD127 within these cells, as it was done for CD8^+^ T cells. One would probably need to pool eyeballs obtained from several dozens of mice into a single sample for an effective performance of such an analysis.

The experiments reported in this paper showed that the mean percentage of CD4^-^CD8^+^ T cells (further referred to as CD8^+^ T cells) within the total population of lymphocytes isolated from AH of mice was as high as 28.69% (± 9.41), while the value of this parameter for PB was 12.76 (± 3.44) (Fig 1A and 1C). Subpopulations with high and low intensity of CD8 expression could be distinguished within these lymphocytes. In this respect, AH CD8^+^ T cells again differed from PB CD8^+^ cells: the mean percentage (relative to the total CD8^+^ lymphocyte population) of CD8^high^ and CD8^low^ cells in AH samples was 34.09 (± 12.00) and 65.91 (± 12.00), respectively, while the corresponding values of this parameter for PB CD8^+^ T cells were 77.51 (± 10.82) and 21.68 (± 11.38) (Fig 1B and 1C). All the above parameters were significantly different between PB and AH samples. In order to make sure that CD8^+^ cells were T lymphocytes, the cells were additionally labeled towards expression of CD3 molecule, i.e. a marker of T cells. This part of the research showed that an average of 90.30% (± 3.78, n = 10) of AH CD8^+^ cells demonstrated expression of CD3, while the value of this parameter for PB CD8^+^ cells was 96.29% (± 2.34, n = 10). Hence, the CD8^+^ cells detected in the eye chambers in mice should be considered as T lymphocytes, although it needs to be borne in mind that about 1/10 of this subpopulation is most evidently composed of some other type of immunocompetent cells.

As mentioned before, the available literature contains hardly any information on the presence of CD4^+^ and CD8^+^ T cells within eye chambers of healthy eyes, although there are some data regarding this issue. Avundruk et al. [16–17] demonstrated that the CD4^+^/CD8^+^ cell ratio in AH was 0.77%, while the value of this parameter in blood was 1.61% [16] or 1.56% [17]. These investigations clearly demonstrate that counts of CD8^+^ T cells in AH are higher than those of CD4^+^ T cells, whereas in PB the relationship is reverse. Thus, there is some congruity between the cited research and our study in this respect, although CD4^+^ T cells in mouse AH constitute only a trace subset. The existence of a body compartment which to a considerable extent is patrolled/homed by CD8^+^ T cells at the trace presence of CD4^+^ T cells seems to be an unusual phenomenon. Most evidently, such selective patrolling/homing of these cells to the eye chambers is engaged in the maintenance of ocular immune privilege. Avunduk et al. [17] postulated that a high concentration of CD8^+^ and a low concentration of CD4^+^ T cells in AH may be one of the contributing factors in the pathogenesis of the immunosuppressive behavior of the anterior chamber. Moreover, the majority of AH CD8^+^ T cells exhibited low expression of CD8, which suggests that these cells may be involved in the induction and maintenance of the immune privilege of the eye, because the down-regulation of CD8 has been postulated as one of the mechanisms for peripheral tolerance [19–21].

**Fig 2:**
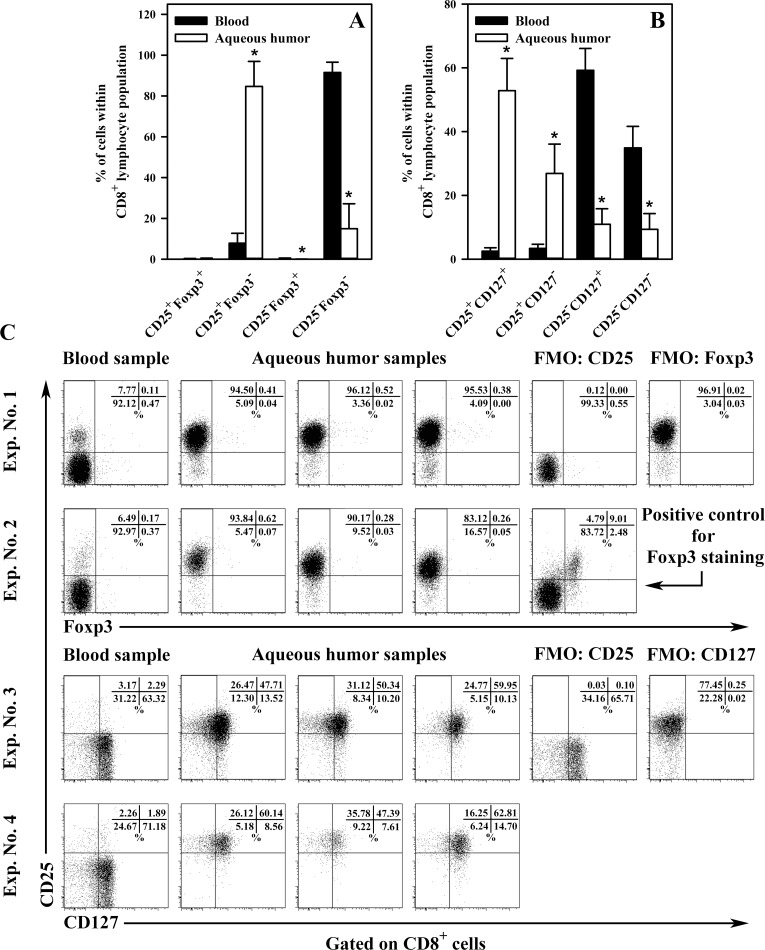
**Expression/co-expression of CD25 and Foxp3 (A) and CD25 and CD127 (B) on CD8**^**+**^ **cells in mouse peripheral blood and aqueous humor.** The results are expressed as a percentage of CD25^+^Foxp3^+^, CD25^+^Foxp3^-^, CD25^-^Foxp3^+^, CD25^-^Foxp3^-^ (A), CD25^+^CD127^+^, CD25^+^CD127^-^, CD25^-^CD127^+^ and CD25^-^CD127^-^ cells (B) within CD8^+^ T lymphocyte population. Results are the mean (± SD) of 3 independent experiments for each type of labeling. Peripheral blood samples were collected from individual mice [n = 15 (A); n = 15 (B)], whereas each aqueous humor sample consisted of cells pooled from the eyeballs of 5 (or sometimes more) mice [n = 15 samples (A); n = 15 samples (B)]. Examples of cytograms (dot plots) from different samples for different experiments (C). On the basis of expression/co-expression of CD25 and Foxp3 or CD25 and CD127, CD8^+^ T cells were subdivided into the following subsets: CD25^+^Foxp3^+^, CD25^+^Foxp3^-^, CD25^-^Foxp3^+^, CD25^-^Foxp3^-^ (panels 1 and 2), CD25^+^CD127^+^, CD25^+^CD127^-^, CD25^-^CD127^+^ and CD25^-^CD127^-^ cells (panels 3 and 4). Fluorescence minus one (FMO) staining was used to confirm the gating strategy used to identify CD25- (panels 1 and 3), Foxp3- (panel 1) and CD127-expressing cells (panel 3). Gated CD4^+^ T cells from peripheral blood served as a positive control for Foxp3 staining (panel 2). *P < 0.001.

The analysis of AH CD8^+^ T cells in terms of the expression/co-expression of CD25 and Foxp3 manifested that on average as many as 84.71% (± 12.21) of these lymphocytes showed expression of CD25 molecule, which was not concomitant with Foxp3 co-expression (Fig 2A, panels 1 and 2 of Fig 2C). Regarding this parameter, AH CD8^+^ cells were drastically different from PB CD8^+^ cells (P < 0,001), because only just 7.85% (± 4.84) of lymphocytes among the latter set displayed the CD25^+^Foxp3^-^ phenotype. Similarly to blood, CD8^+^CD25^+^Foxp3^+^ cells in AH constitute a very small subpopulation because the average percentage of CD25^+^Foxp3^+^ cells among CD8^+^ cell subset from AH and PB samples was just 0.32 (± 0.21) and 0.21 (± 0.14), respectively (Fig 2A, panels 1 and 2 of Fig 2C); these values did not differ significantly No presence of cells with the CD8^+^CD25^-^Foxp3^+^ phenotype in AH samples was detected (Fig 2A, panels 1 and 2 of Fig 2C).

CD25 is α chain of the IL-2 receptor (IL-2R) that is expressed on Tregs and activated T and B lymphocytes, whereas Foxp3 is a unique marker and a “master” regulator of the development and suppressive function of CD4^+^ Treg cells [2]. Foxp3 currently represents the most specific marker used to distinguish Treg cells (CD4^+^CD25^+^Foxp3^+^) from their thymic progenitors and activated effector T cells (CD4^+^CD25^+^Foxp3^-^); the CD25-non-expresing T cells represent resting cells, i.e. naive and memory T cells. Assuming that Foxp3 confers suppressive properties and is confined to regulatory T cells, the results obtained indicate that CD8^+^ cells with the regulatory phenotype, i.e. CD8^+^CD25^+^Foxp3^+^ cells, occur naturally in the anterior eye segment of healthy mice, although this subset is very small. In 2003, Cosmi et al. [22] were the first to identify the existence of a subset of human CD8^+^CD25^+^Foxp3^+^ thymocytes sharing a phenotype, functional features, and mechanism of action with CD4^+^CD25^+^Foxp3^+^ Tregs. The results of research by Correale and Villa [23] confirmed the existence of such cells in humans and indicated that defects in their function can cause induction of autoimmune reactions. The presence of natural regulatory CD8^+^CD25^+^Foxp3^+^ T cells has also been demonstrated in mice [24]. There are reports available suggesting that cells with the CD8^+^CD25^+^Foxp3^+^ phenotype play an important role in the immune regulation of the anterior segment of an eye [5] and in the development of ACAID [6–7]. However, it should be emphasized that these studies described inducible but not naturally-occurring CD8^+^CD25^+^Foxp3^+^ T cells, and they were generated under *in vitro* conditions rather than investigated for their presence in the eye.

Basically, extrathymic Foxp3-non-expressing CD25-postive T cells should be treated as activated effector T cells. A critical point in the development of an immune response is the activation of T lymphocytes that requires IL-2 binding to its high-affinity IL-2R (i.e. CD25) for optimal signalling. The study showed that nearly 85% AH CD8^+^ T cells expressed CD25 without concomitant Foxp3 expression. In the light of the above, these cells should be considered as activated effector CD8^+^ T cells. However, such interpretation is naturally unacceptable because the animals used in the experiment were healthy and had never been immunized. Thus, subsequent tests were carried out concerning the status of these cells, in which the expression of the CD127 antigen, which is the α-chain of the IL-7 receptor, was assessed. CD127 is expressed by naive and long-lived memory CD8^+^ T cells [25], while being down-regulated on activated effector CD8^+^ T cells [26]. The experiments showed that as many as 52.84% (± 10.12) of AH CD8^+^ T cells co-expressed CD25 and CD127, whereas 26.89 (± 9.19) and 10.91 (± 4.90) of these cells exhibited, respectively, the CD25^+^CD127^-^ and CD25^-^CD127^+^ phenotypes (Fig 2B, panels 3 and 4 of Fig 2C). 9.36% (± 4.92) of AH CD8^+^ T cells did not demonstrate the expression of either CD25 or CD127. With respect to PB CD8^+^ T cells, co-expression of these two molecules followed a completely different pattern because 59.21% (± 6.86) of cells from this population expressed CD127 but not CD25; only 2.49% (± 1.06) PB CD8^+^ T cells co-expressed CD25 and CD127 (Fig 2B, panels 3 and 4 of Fig 2C). All the above parameters were significantly different between PB and AH samples. Thus, 63.75% of AH CD8^+^ T cells expressed CD127, which suggests that they are not activated effector cells, but that this subpopulation most clearly represents naive T lymphocytes. It is worth noting that AH CD8^+^ T cells are very similar to PB CD8^+^ T cells with regard to the percentage of CD127 expressing cells because the average expression of CD127 within these lymphocytes was 61.70%. As mentioned earlier, although numerous reviews and textbooks state that naive T cells do not normally enter non-lymphoid organs, several studies demonstrated that as part of the normal migratory pathway, these cells enter non-lymphoid organs [8–13]. The results obtained in our research suggest that eye chambers in mice belong to the normal migratory pathway of naive CD8^+^ T cells, whereas their phenotype (in respect of the presence of CD25 molecule and intensity of CD8 expression) changes under the influence the microenvironment of the anterior segment of the eye.

It is extremely intriguing why about ¼ of AH CD8^+^ cells display the CD25^+^Foxp3^-^CD127^-^ phenotype. In theory, such a phenotype should be equated with activated effector T cells, but for the reasons explained previously this possibility ought to be excluded. It can be hypothesized that these cells are progenitors of inducible CD8^+^CD25^+^Foxp3^+^ Tregs. It is now known that thymic CD4^+^CD25^+^Foxp3^-^ T cells represent progenitors of nTregs [27]. Most CD4^+^CD25^+^Foxp3^+^ Treg precursors are newly formed thymocytes that acquire Foxp3 expression on antigen encounter in the thymus. Differentiation of Tregs, however, can also occur in the periphery [28]. Importantly, it is well established that CD127 expression is down-regulated not only on activated effector T cells, but also on CD4^+^CD25^+^Foxp3^+^ Tregs [29]. Considering above and certain implications suggesting that CD8^+^CD25^+^Foxp3^+^ Tregs can be induced within the anterior part of the eye [5], the hypothesis presented above does not seem groundless.

The sudies suggest that most of AH CD8^+^ T cells exhibit constitutive expression of CD25. This finding seems peculiar because to the best of our knolwedge extraocular CD8^+^ T cells in healthy animals express CD25 in a very limited extent, which is actually supported by the results regarding the percentage of CD25 expressing cells within PB CD8^+^ T cell subset. Further investigations are required to elucidate the meaning of this phenomenon.

In conclusion, the conducted research revealed that CD8^+^ T cells are normally present in mouse eye chambers, but in contrast to PB CD8^+^ T cells, most of these cells display low expression of CD8. Within this population, the presence of cells with the regulatory phenotype, i.e. CD25^+^Foxp3^+^ cells, was determined although this subset, similarly to its phenotypic counterpart in PB, is very small. Likewise, in terms of the expression of CD127, AH CD8^+^ T cells do not differ substantially from PB CD8^+^ T cells. In turn, AH CD8^+^ T cells are an exceptional population with regard to the expression of CD25 because in nearly 85% of cases they express this molecule without concomitant Foxp3 expression. Despite displaying such a phenotype, these cells should not be considered as activated cells because most of them co-express CD127, which indicates that they are naive lymphocytes. In the context of the markers used in our study, CD8^+^CD25^+^CD127^+^Foxp3^-^ T cells represent the most numerous subset of AH CD8^+^ T cells. The results achieved in our study suggest that eye chambers in mice are part of the normal migratory pathway of naive CD8^+^ T cells. CD4^+^ cells are only a trace subset among the whole AH lymphocyte population. Most evidently, such selective and specific homing of CD8^+^ and CD4^+^ cells to the eye chambers is involved in the induction and maintenance of ocular immune privilege.
